# Metrological framework to support accurate, reliable, and reproducible nucleic acid measurements

**DOI:** 10.1007/s00216-021-03712-x

**Published:** 2021-11-04

**Authors:** Mojca Milavec, Megan H. Cleveland, Young-Kyung Bae, Robert I. Wielgosz, Maxim Vonsky, Jim F. Huggett

**Affiliations:** 1 Department of Biotechnology and Systems Biology, National Institute of Biology, Večna pot 111, 1000 Ljubljana, Slovenia; 2 National Institute of Standards and Technology, 100 Bureau Drive, Gaithersburg, MD 20899 USA; 3 Korea Research Institute of Standards and Science (KRISS), Daejeon, Republic of Korea; 4 Bureau International Des Poids Et Mesures (BIPM), Pavillon de Breteuil, 92312 Sèvres Cedex, France; 5 D.I. Mendeleev Institute for Metrology, Moskovsky pr., 19, Saint-Petersburg, 190005 Russian Federation; 6 National Measurement Laboratory (NML), LGC, Queens Road, Teddington, TW11 0LY Middlesex UK; 7 School of Biosciences & Medicine, Faculty of Health & Medical Science, University of Surrey, Guildford, UK

**Keywords:** Nucleic acids (DNA | RNA), Measurements, Accuracy, Traceability

## Abstract

Nucleic acid analysis is used in many areas of life sciences such as medicine, food safety, and environmental monitoring. Accurate, reliable measurements of nucleic acids are crucial for maximum impact, yet users are often unaware of the global metrological infrastructure that exists to support these measurements. In this work, we describe international efforts to improve nucleic acid analysis, with a focus on the Nucleic Acid Analysis Working Group (NAWG) of the Consultative Committee for Amount of Substance: Metrology in Chemistry and Biology (CCQM). The NAWG is an international group dedicated to improving the global comparability of nucleic acid measurements; its primary focus is to support the development and maintenance of measurement capabilities and the dissemination of measurement services from its members: the National Metrology Institutes (NMIs) and Designated Institutes (DIs). These NMIs and DIs provide DNA and RNA measurement services developed in response to the needs of their stakeholders. The NAWG members have conducted cutting edge work over the last 20 years, demonstrating the ability to support the reliability, comparability, and traceability of nucleic acid measurement results in a variety of sectors.

**Supplementary Information:**

The online version contains supplementary material available at 10.1007/s00216-021-03712-x.

## Introduction

Nucleic acid (NA) analysis is fundamental for many areas of life sciences including biotechnology, cell biology, genetics, microbiology, and molecular biology. Applied nucleic acid analysis is increasingly required for applications in different fields such as medicine, pharmacy, veterinary medicine, food safety, and environmental monitoring. Nucleic acid analysis encompasses the detection, identification, and quantification of nucleic acids from different organisms, often from diverse matrices. Accurate and metrologically traceable measurements of nucleic acids enable reliable results to support decision makers like healthcare workers, sanitary inspectors, and competent authorities.

In the last few decades, nucleic acid analysis has rapidly developed supported by an increasingly diverse array of technologies. After the understandable slow beginnings of RNA sequencing in the 1960s, DNA sequencing in the 1970s, and the invention of polymerase chain reaction (PCR) in the 1960s, all these analytical approaches faced extremely intensive developments. Currently, RNA sequencing is an indispensable tool for transcriptome-wide analysis and is contributing to a fuller understanding of RNA biology [1]; third-generation DNA sequencing has revolutionized sequencing by increasing the length of reads and reducing the time and costs of analysis [2, 3]; and digital PCR (dPCR) has enabled absolute quantification of specific nucleic acid targets [4].

Despite broad use of nucleic analysis, comparability of nucleic acid quantification results is unsatisfactory among laboratories in many areas. This is especially evident where quantitative analysis of nucleic acids is required. For example, in the food and feed safety area, national reference laboratories and official control laboratories in the European Union use common validated methods to determine the mass fraction of the genetically modified organisms (GMOs) in samples. Reports on laboratory performances in the proficiency tests provided by the European Union Reference Laboratory for Genetically Modified Food and Feed (EURL-GMFF) [5] usually show up to fivefold and occasionally up to tenfold differences among laboratory results [6, 7]. Additionally, in External Quality Assessment schemes, results from participating laboratories can vary by more than 100-fold [8, 9].

It is unfortunate that metrology, the science of measurement, and metrological considerations (such as routes to traceability and sources of uncertainty) are sometimes overlooked in a number of applied areas, with those applying nucleic acid analysis typically assuming their systems are fit for purpose in this respect. Certainly, many breakthroughs in sequence discovery, genome evaluation, and routine monitoring have succeeded without the assistance of the measurement science community. Yet as these fields have pushed to offer ever more high-throughput (“omics”) and precise quantitative solutions, they have been met with challenges associated with standardization and traceability of measurement results. Many of those working in these spaces neither realize that measurement science is a field in its own right, nor that a global infrastructure exists to support them in this respect. Given the wide variety of applications in the nucleic acid analysis space, there are numerous examples where applying measurement science could support improving the routine application both in basic research and applied scenarios. This overview outlines the benefits of metrological support for nucleic acid analysis in the life science sectors that are more metrologically established, while also championing advancements in the other sectors.

## NAWG at CCQM

Many international organizations and societies are making efforts to improve the consistency of results among laboratories in general such as the International Organization for Standardization (ISO) [10] or the European Committee for Standardization (Cen) [11] and in specific fields like healthcare, e.g., the World Health Organization (WHO) [12], the Joint Committee for Traceability in Laboratory Medicine (JCTLM) [13], the International Federation of Clinical Chemistry and Laboratory Medicine (IFCC) [14], the Standardisation of Genome Amplification Techniques (SoGAT) [15], and the European Society of Clinical Microbiology and Infectious Diseases (ESCMID) [16]; or food safety and security in the field of detection, identification, and quantification of genetically modified organisms, e.g., the European Network of GMO Laboratories (ENGL) [17] and the Codex Alimentarius [18]; or in plant health, e.g., the International Plant Protection Convention (ICPP) [19] and the European and Mediterranean Plant Protection Organization (EPPO) [20]. Short descriptions of listed organizations, bodies, and societies are in Table 1.

**Table 1:** List of abbreviations and short descriptions of relevant metrology and standardization/harmonization bodies, associations, organizations, consortia, and programs

Abbreviation	Short description				Reference
*Metrology bodies, organizations, consortia, and programs*					
APMP	**Asia Pacific Metrology Programme** is a group of NMIs from the Asia–Pacific region engaged in improving regional metrological capability through the sharing of expertise and exchange of technical services among member laboratories. APMP is also a RMO recognized by the CIPM for the purpose of worldwide mutual recognition of measurement standards and of calibration and measurement certificates				[21]
BAWG	**BioAnalysis Working Group** was established in 2001 and dedicated to the emerging field of metrology in biology. Due to the increasing and more specialized activities, BAWG was split into three new CCQM working groups: CAWG, NAWG, and PAWG				[22]
BIPM	**International Bureau of Weights and Measures (*French*: Bureau International des Poids et Mesures)** is the intergovernmental organization in which governments cooperate on matters of metrology and measurement standards				[23]
CAWG	**Cell Analysis Working Group** of the CCQM is an international group dedicated to improving the global comparability of cell measurements, including identification and quantification of intact cells and cell properties indicative of function in complex matrices and mixtures				[24]
CCQM	**Consultative Committee for Amount of Substance: Metrology in Chemistry and Biology** is responsible for developing, improving, and documenting the equivalence of national standards (certified reference materials and reference methods) for chemical and biological measurements. It advises the CIPM on matters related to chemical and biological measurements, including advice on the BIPM scientific program activities				[25]
CGPM	**General Conference for Weights and Measures** is the supreme body of BIPM. The CGPM is composed of delegates of the governments of the member states and observers from the associates of the CGPM. Under its authority, the CIPM exercises exclusive direction and supervision of the BIPM				[26]
CIPM	**International Committee for Weights and Measures** is promoting worldwide uniformity in units of measurement and does so through direct action or by submitting draft resolutions to the CGPM				[27]
CIPM MRA	**CIPM Mutual Recognition Arrangement** is the framework through which NMIs demonstrate the international equivalence of their measurement standards and the calibration and measurement certificates they issue				[28]
CMC	Under a **Calibration and Measurement Capability**, the measurement or calibration should be (a) performed according to a documented procedure and have an established uncertainty budget under the management system of the institute; (b) performed on a regular basis (including on demand or scheduled for convenience at specific times in the year); and (c) available to all customers				[29]
COOMET	**Euro-Asian Cooperation of National Metrological Institutions** is a joint forum of metrologists of the Euro-Asian region, a steadily and effectively working regional metrology organization. Cooperation within COOMET and its results allow its member countries to successfully solve metrological issues, which national economies face under the conditions of market globalization				[30]
DI	**Designated Institute** is operating at the top of the national metrology system like NMI, complementing the fields of activities of the NMI and bringing in expertise in metrological areas not covered by the NMI				/
EMRP	**European Metrology Research Programme** (2009–2013) has enabled European metrology institutes, industrial organizations, and academia to collaborate on joint research projects within specified fields: industry, energy, environment, health, new technologies, and SI units				[31]
EMPIR	**European Metrology Programme for Innovation and Research** (2014–2020) coordinated research projects to address grand challenges, while supporting and developing the SI system of units of measurement. There was an increased focus on innovation activities to address industry needs and accelerate the uptake of research outputs. The capacity-building projects of the program aimed at bridging the gap between EU member states with emerging measurement systems and those with more developed capabilities				[32]
ERCC	The aim of the **External RNA Controls Consortium** is to build the measurement assurance tools needed to support reproducible gene expression measurements. ERCC partners from industry, government, and academia develop RNA spike-in controls and establish analytical methods for bringing reproducible gene expression measurements into routine, high-quality practice				[33]
EURAMET	**European Association of National Metrology Institutes** is the RMO of Europe. EURAMET coordinates the cooperation of NMIs in Europe in fields such as research in metrology, traceability of measurements to the SI units, international recognition of national measurement standards, and related CMCs. Through knowledge transfer and cooperation among the members, EURAMET facilitates the development of the national metrology infrastructures				[34]
KCDB	**Key Comparisons Database** is a freely available web resource related to the CIPM MRA. The outputs of CIPM MRA are the internationally recognized (peer-reviewed and approved) CMCs of the participating institutes. Approved CMCs and supporting technical data are publicly available from the KCDB				[35]
MWG 8	**Chemical Metrology Working Group** in SIM supports SIM and its member NMIs/DIs in reaching the obligations and requirements of CIPM-MRA in the field of metrology in chemistry and biology measurements				[36]
NAWG	**Nucleic Acid Analysis Working Group** of the CCQM is an international group dedicated to improving the global comparability and metrological traceability of measurement results for the analysis of nucleic acid polymer sequences, their modifications, and their abundance				[37]
NMI	**National Metrology Institute** is the national authority on measurement. National metrology institutes have the responsibility of maintaining national measurement standards and disseminating the international system of units nationally				/
PAWG	**Protein Analysis Working Group** of the CCQM is an international group dedicated to improving the global comparability of protein measurements, including (a) development and validation of reference measurement procedures for purity assessment of high-purity peptide and protein materials suitable for calibration standards and (certified) reference materials; (b) qualitative and quantitative analyses of peptides and proteins in complex biological matrices and biopharmaceuticals; (c) other more specialized measurements related to proteins such as catalytic enzymatic activities, as well as biotherapeutic and antibody characterization				[38]
RMO	**Regional metrology organizations** are regional associations of national metrology institutes. They play an important role in the CIPM MRA as they (a) make proposals to the consultative committees on the choice of key comparisons, (b) carry out the RMO key comparisons, described in the Technical Supplement to the CIPM MRA, corresponding to the CIPM key comparisons; (c) participate in the joint committee of the regional metrology organizations and the BIPM; and (d) carry out supplementary comparisons and other actions designed to support mutual confidence in the validity of calibration and measurement certificates issued by participating institutes				/
SI	**International System of Units** is the recommended practical system of units of measurement				[39, 40]
SIM	**Inter-American Metrology System** was created to promote international, and regional, and particularly Inter-American cooperation in metrology issues and is committed to implementing a global measuring system upon which all users may rely. SIM promotes and supports an integrated measurement infrastructure in the Americas, which enables each member NMI to stimulate innovation, competitiveness, trade, consumer safety, and sustainable development by effectively participating in the international metrology community				[41]
TC-MC	**Technical Committee of Metrology in Chemistry** is a Joint EURAMET-Eurachem Technical Committee. It is concerned with primary methods and reference materials for chemical measurements and research in metrology to support different sectors in chemistry. Nucleic acid analysis activities are under the responsibility of the SubCommittee Bio and Organic Analysis				[42]
TCQM	**Technical Committee Amount of Substance** of APMP is responsible for developing and improving the equivalence of national reference systems for chemical and biological measurements. The TCQM is also responsible for initiating and monitoring the TCQM comparison programs and ensuring that these link to the international comparison programs conducted through the CIPM CCQM				[21]
*Standardization/harmonization bodies and organizations*					
Cen	**European Committee for Standardization** is an association that brings together the National Standardization Bodies of 34 European countries				[11]
Codex Alimentarius	The **Codex Alimentarius** is a collection of internationally adopted food standards and related texts presented in a uniform manner. These food standards and related texts aim at protecting consumers’ health and ensuring fair practices in the food trade. The publication of the Codex Alimentarius is intended to guide and promote the elaboration and establishment of definitions and requirements for foods to assist in their harmonization and in doing so to facilitate international trade				[18]
ESCMID	**European Society of Clinical Microbiology and Infectious Diseases** is a non-profit organization whose mission is to improve the diagnosis, treatment, and prevention of infection-related diseases. This is achieved by promoting and supporting research, education, training, and good medical practice				[16]
EURL-GMFF	**European Union Reference Laboratory for Genetically Modified Food and Feed** is responsible for the scientific assessment and validation of detection methods for genetically modified food and feed as part of the European Union authorization procedure and provides support to the National Reference Laboratories for control of GMOs in the European Union member states				[5]
ENGL	**European Network of GMO Laboratories** plays an eminent role in the development, harmonization, and standardization of means and methods for sampling, detection, identification, and quantification of genetically modified organisms (GMOs) in a wide variety of products, ranging from seeds, grains, to food and feed stuff				[17]
EPPO	**European and Mediterranean Plant Protection Organization** is an intergovernmental organization responsible for cooperation in plant health within the Euro-Mediterranean region. Its objectives are to protect plants, by developing international strategies against the introduction and spread of pests which are a threat to agriculture, forestry, and the environment, and by promoting safe and effective pest control methods. EPPO is a standard-setting organization which has produced a large number of standards in the areas of plant protection products and plant quarantine				[20]
ICPP	**International Plant Protection Convention** is an intergovernmental treaty signed by over 180 countries, aiming to protect the world’s plant resources from the spread and introduction of pests, and promoting safe trade. The convention introduced international standards for phytosanitary measures as its main tool to achieve its goals, making it the sole global standard setting organization for plant health				[19]
IFCC	**International Federation of Clinical Chemistry and Laboratory Medicine** is a worldwide, non-political organization for clinical chemistry and laboratory medicine. As such, it has a range of roles that include (1) global standard setting in collaboration with other international organizations, (2) supporting its members through scientific and educational endeavor, and (3) providing a series of congresses, conferences and focused meetings in order for laboratory medicine specialists to meet and present original findings and best practice				[14]
ISO	**International Organization for Standardization** is an independent, non-governmental international organization developing and publishing international standards				[10]
JCTLM	**Joint Committee for Traceability in Laboratory Medicine** is an international consortium that promotes the global standardization of clinical laboratory test results, and provides information on reference materials, reference measurement methods, and services that are available from around the world				[13]
SoGAT	**Standardisation of Genome Amplification Techniques** is an international working group dedicated to the standardization of nucleic acid amplification technology–based tests for human infectious diseases				[15]
WHO	**World Health Organization** is the United Nations agency that connects nations, partners, and people to promote health. WHO is dedicated to the well-being of all people and guided by science leads and champions global efforts to give everyone, everywhere an equal chance to live a healthy life				[12]
*Other relevant abbreviations*					
CRM	**Certified reference material** is reference material, accompanied by documentation issued by an authoritative body and providing one or more specified property values with associated uncertainties and traceabilities, using valid procedures				[40]
dPCR	**Digital polymerase chain reaction** is advanced PCR enabling quantification of a specific region of a nucleic acid				
GMOs	**Genetically modified organisms** are animals, plants, or microbes whose genetic material has been changed using techniques of modern biotechnology called gene technology or genetic engineering				
NIPT	**Non-invasive prenatal testing**				
PCR	**Polymerase chain reaction** is a common molecular biology technique used to produce multiple copies of a specific region of DNA				
RM	**Reference material** is a material sufficiently homogeneous and stable with reference to specified properties, which has been established to be fit for its intended use in measurement or in examination of nominal properties				[40]

One of the international groups dedicated to improving the comparability of nucleic acid measurements independent of the field is the Working Group on Nucleic Acid Analysis (NAWG) [37] of the Consultative Committee for Amount of Substance: Metrology in Chemistry and Biology (CCQM) [25]. CCQM is one of the consultative committees (CCs) of the International Committee for Weights and Measures (CIPM) [27] with a mission to advance global comparability of chemical and biological measurement standards and capabilities, enabling member states and associates to make measurements with confidence, and with a published strategy for 2021–2030 [43] to do so. Among its activities, CCQM has responsibility for developing, improving, and documenting the equivalence of National Metrological Institutes (NMIs) and Designated Institutes (DIs) calibration and measurement capabilities, national standards (certified reference materials and reference methods) for chemical and biological measurements, following the processes set out in the CIPM Mutual Recognition Arrangement (CIPM MRA) [28]. More information on the bodies mentioned above is in Table 1.

Activities related to nucleic acid analysis within CCQM began in 1999, the year that the XXI General Conference for Weights and Measures (CGPM) [26] discussed the growing importance of biotechnology in human health, food production, forensic medicine, and the protection of the environment. CCQM recognized the need for accurate, SI-traceable measurements in these fields and the lack of an adequate metrological infrastructure to ensure such traceability. It recommended that national laboratories consider developing programs related to the measurement of quantities important in biotechnology and that national laboratories collaborate with international scientific unions and organizations to establish an adequate international measurement infrastructure to ensure traceability to the SI in measurements in biotechnology.

An Ad Hoc Biometrology Task Group was established, and in 2000, it presented a report on new measurement challenges. In 2001, this task group became the BioAnalysis Working Group (BAWG) of the CCQM [22]. In 2014, the BAWG was split into three new CCQM working groups according to major analytes: the Nucleic Acid WG – NAWG [37], the Protein Analysis WG – PAWG [38], and the Cell Analysis WG – CAWG [24] due to the growing number of separated activities and increased membership. In the same year, the name and the scope of the CCQM were broadened from CCQM—Metrology in Chemistry to CCQM—Metrology in Chemistry and Biology [25]. Short descriptions of working groups are in Table 1.

The focus of the NAWG is to support global comparability and metrological traceability of measurement results in the area of the analysis of nucleic acid polymer sequences, their modifications, and their abundance. Members of the NAWG are NMIs and DIs that provide nucleic acid measurement services developed in response to the needs of their national and international stakeholders. The ubiquity of the nucleic acid analysis methods, the molecules they target, and the associated challenges are agnostic to sectors in most cases. Consequently, as an international working group that focuses on nucleic acid analysis regardless of sector, the NAWG is uniquely placed to advance the measurements in one sector using knowledge from another [44]. This constitutes a firm basis for NAWG’s ability to respond to challenges in the nucleic acid analysis field despite the field’s rapid technological development and implementation. This was exemplified by the response of the NAWG to the COVID-19 pandemic through the SARS-CoV-2 pilot study (CCQM-P199.b) with the participation of 18 NMIs/DIs, many of whom had not previously measured viral genomic material [45]. The NAWG will need to explore how this pan sector characteristic can be capitalized on to support different groups of stakeholders in the future.

## Regional metrology organizations

Regional metrology organizations (RMOs) also organize activities for metrology in nucleic acid analysis. In the Asia Pacific Metrology Programme (APMP) [21], the Technical Committee Amount of Substance (TCQM) is responsible for developing and improving the equivalence of national reference systems for chemical and biological measurements. While the activities of the TCQM involve quantification of microorganisms in drinking water and food, the quantification is currently not based on nucleic acid measurement but on classical microbiology and culture-based methods.

In the Euro-Asian Cooperation of National Metrological Institutions (COOMET) [30], the bioanalysis subcommittee was established under the Technical Committee 1.8 “Physic-Chemistry” [46] in 2019 and the importance of genetic technologies and reference material development to ensure the traceability of bioanalytical measurements was noted. While a pilot interlaboratory comparison on quantitative determination of human DNA is ongoing, the first bioanalysis subcommittee meeting is expected to be held in 2021 and its activity will be aligned with the NAWG strategy.

Within the Inter-American Metrology System (SIM) [41], the field of metrology in chemistry and biology measurements is the responsibility of the Chemical Metrology Working Group 8 (MWG 8) [36]. MWG 8 provides tools to guarantee the traceability and reliability of measurement results from environmental monitoring to health care, including food quality and food safety. It also offers support in fundamental research ranging from material science (nanotechnology) to bioscience and biotechnology, fair trade, and innovation.

EURAMET is the European Association of National Metrology Institutes [34]. Nucleic acid analysis activities are the responsibility of the Technical Committee of Metrology in Chemistry (TC-MC), SubCommittee Bio and Organic Analysis [42]. In addition, European NMIs and DIs work closely together with stakeholders through the European Metrology Research Programme (EMRP) [31] and European Metrology Programme for Innovation and Research (EMPIR) [32]. Notable projects with relevance to nucleic acid analysis include “Metrology for monitoring infectious diseases, antimicrobial resistance, and harmful micro-organisms” (Infect-Met, HLT08) [47], “Novel materials and methods for the detection, traceable monitoring and evaluation of antimicrobial resistance” (AntiMicroresist, 15HLT07) [48], “Traceability for biologically relevant molecules and entities” (BioSITrace, SIB54) [49], and “Metrology to enable rapid and accurate clinical measurements in acute management of sepsis” (SEPTIMET, 18HLT03) [50]. Furthermore, the European Metrology Network for Traceability in Laboratory Medicine (EMN TraceLabmed) [51] brings stakeholders such as proficiency testing providers, in vitro diagnostics manufacturers, regulators, and NMIs/DIs together to address stakeholder needs, which is especially important in the light of the new European IVD regulation [52, 53].

## Standardization, traceability, and scientific challenges

The primary focus of NAWG is to support the development and maintenance of measurement capabilities and dissemination of measurement services from NMIs/DIs. This is supported via interlaboratory studies to evaluate the competencies of NMIs and DIs and provide evidence of the validity and international equivalence of measurement services for customers and stakeholders worldwide. Based on their performance in the interlaboratory studies, NMIs and DIs can submit their calibration and measurement capabilities (CMCs) for review. Following the successful review by RMOs and designated CCQM members, CMCs are entered into the International Bureau of Weights and Measures (BIPM) [23] key comparison database (KCDB) [29, 35]. CMCs are characterized by the measured quantity and associated measurement uncertainty (generally given at a 95% level of confidence) for a given range, the method or instrument used, the values of influencing parameters, and any other relevant information [29]. In addition to supporting CMCs, NAWG is using these studies to underpin the development of reference measurement systems and to establish the traceability of measurements to the International System of Units [39].

In parallel with recognizing current and future needs in standardization and traceability, NAWG is also addressing some of the fundamental scientific challenges associated with nucleic acid analysis. Nucleic acids vary in their stability which can impact both reference materials and biobanks, as well as routine testing during sample handling, isolation, and purification. DNA is generally considered stable; however, low mass concentrations (less than 5 ng/μL) of both DNA and RNA may require the addition of stabilizing “carrier” molecules (such as yeast tRNA or other background heterologous RNA/DNA) to prevent decreases in concentration over time. RNA can be less stable than DNA, both due to inherent chemical reasons and because RNases, which can quickly degrade RNA, can be both ubiquitous and stable in the environment. Reduced stability of an analyte (in reference materials or clinical samples) may impact the quantity of a given sequence or the composition of its matrix, both of which may affect the measurement result. Furthermore, information on the wider target sequence composition may also be useful as factors like fragment size may also affect stability, resulting in potential discrepancies between methods [54, 55].

Sequence, the order of nucleotide monomers in the polymeric nucleic acid single-strand or double-strand molecule, is a nominal property. Sequence value has no size or magnitude; it is established by what is called an examination procedure in metrology [40, 56]. Sequences, obtained by nominal property value examinations, are deposited to genetic databases. The International Nucleotide Sequence Database Collaboration (INSDC) [57] was founded by GenBank [58], European Nucleotide Archive (ENA) [59], and DNA Databank of Japan (DDBJ) [60]. Since 2002, INSDC maintains the reference sequence database RefSeq [61], which includes the most well-characterized nucleotide and amino acid sequences, providing reference nominal property values for description of target sequences. While sequence examination offers different challenges in terms of standardization to quantitative measurement, there are ongoing activities where NMIs are supporting such approaches, such as in human genomics and metagenomics, as well as work highlighting sources of process error, such as bioinformatic pipelines. It is likely that metrology institutes and molecular reference laboratories will be able to support routine application of such approaches by better characterizing sources of sequence error.

**Fig. 1:**
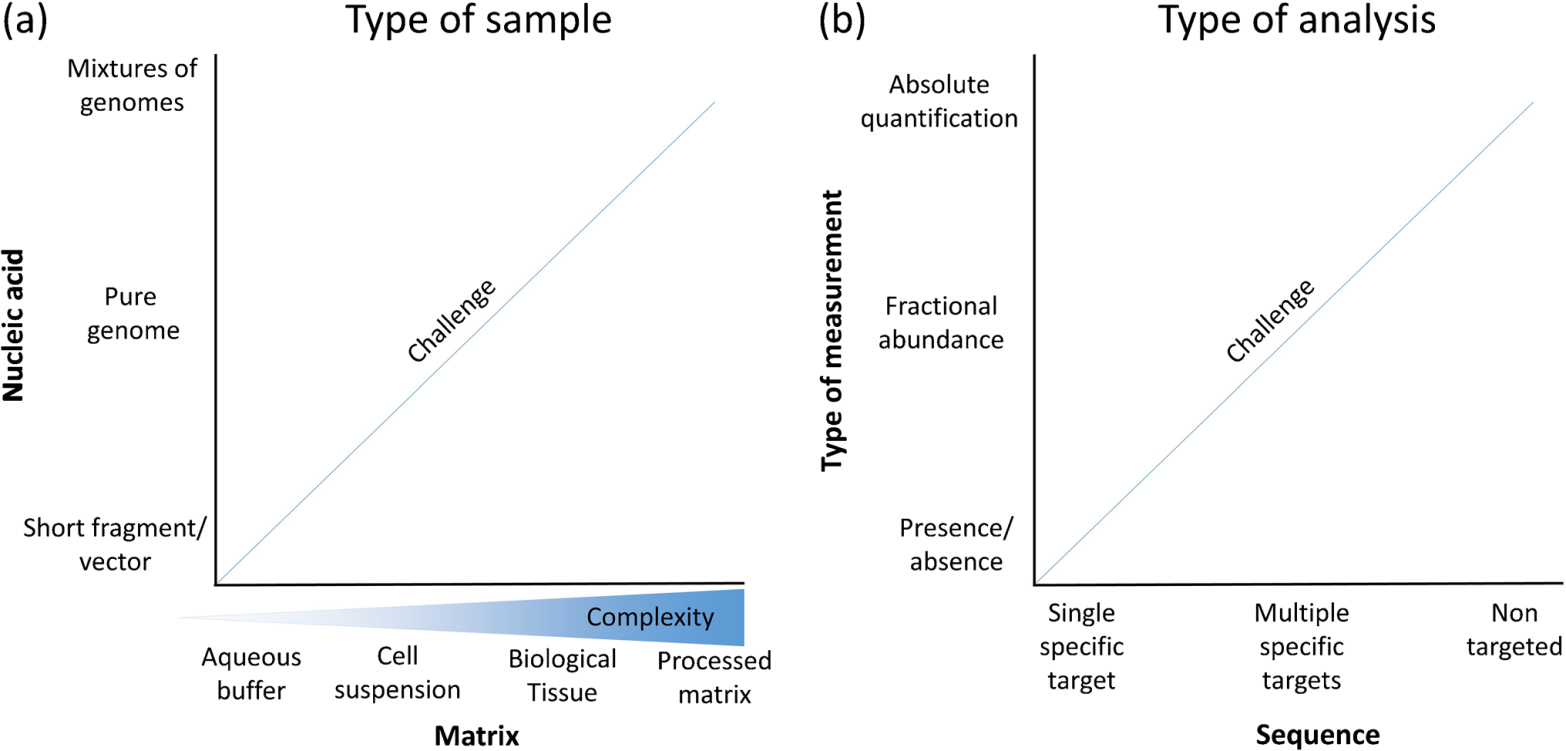
Type of sample (**a**) and type of analysis (**b**); nucleic acid measurements/examinations vary in the complexity of the analysis and the type of sample. The simplest type of measurements would be the detection of the presence or absence of a plasmid in a buffered solution. A much more complex measurement would be the identification of all nucleic acid species in a processed food matrix, along with the abundance of each

Scientific challenges associated with the measurement of a given nucleic acid vary depending on the complexity of the nucleic acid in question (e.g., sequence, size, secondary structure, etc.) as well as the complexity of the matrix in which it has been presented, as nucleic acids are almost exclusively measured in solution. Consequently, considerations associated with sources of uncertainty and routes to traceability frequently include volume measurements as an additional factor. Nucleic acid analyses typically require multistep processes with prior manipulation of the sample to isolate and purify the nucleic acids for further analysis (“pre-analytical” steps). When conducting nucleic acid analysis, a key distinction is whether the analyte is a part of a larger molecule (typically the case with genes associated with genetic modification or pathogen genome) or whether it is contained within the sequence being measured (such as when measuring actionable cancer genetic variants or genetic polymorphism). From an analytical point of view, these two categories can present distinct challenges when considering sources of uncertainty. Furthermore, the analytical sources of error can differ when the same nucleic acid sequence is measured in different matrices (pure nucleic acids in aqueous solution, synthetic matrices, or biological samples) (Fig. 1a).

When discussing a particular measurement/examination, and associated challenges, the type of sample (including the properties of the nucleic acid and its matrix) must also be considered (Fig. 1a). At the simplest level of complexity, nucleic acids can vary from less than 100 to billions of bases (the term is used to describe a nucleotide monomer that is about 300 g/mol; consequently large DNA molecules can be > 10^8^ g/mol). Additionally, samples can consist of mixtures of different genomes, which further complicates measurement/examination. Further potential sources of error are added when additional steps are required to prepare nucleic acids for analysis, such as purification, nucleic acid digestion (e.g., restriction enzyme treatment), adaptation (e.g., adapter ligation), modification (bisulfite conversion), or reverse transcription (generation of complementary nucleic acids). Extraction (purification) is often required when performing nucleic acid analysis, because the matrix is typically unsuitable for the final molecular analysis steps. All these steps contribute to the uncertainty associated with the final analysis.

When considering a given analyte, the type of nucleic acid analysis (Fig. 1b) can be categorized into “targeted” methods (such as PCR or hybridization) or “non-targeted” methods (such as sequencing). Targeted methods identify defined sequence(s) (and potentially their quantities and/or modifications), while with non-targeted methods, the nucleic acid sequence(s) (and potentially their quantities and/or modifications) is unknown and the analysis intends to determine this. The type of analysis can also be broadly categorized into presence/absence “nominal property” examination (e.g., sequence is present or not), measurements of relative quantity/fractional abundance (e.g., percentage of sequence), or absolute quantification (e.g., copy number of sequence per unit volume).

The NAWG has conducted leading work in nucleic acid analysis, demonstrating its ability to support the reliability, comparability, and traceability of nucleic acid measurement results. NAWG had dedicated extensive efforts to understanding and evaluating the absolute quantification of nucleic acid with digital PCR (dPCR). For example, NAWG has investigated the influence of the partition volume on the absolute quantification of DNA in terms of copy numbers [62–65] and also demonstrated that the accuracy of dPCR measurement enables traceability to the International System of Units (SI) for copy number unit 1 through counting [66]. In addition, several members of NAWG were involved in the development of guidelines on dPCR [67, 68] which aims to support researchers in standardizing their experimental protocols and reporting. Besides investigating and discussing the above-described common and general considerations in nucleic acid analysis, NAWG and its members are developing their measurement capacities in specific, stakeholder-driven fields like health and food safety.

Multiple NAWG members were involved in the development of guidelines on standardization for applying nucleic acid analysis. These guidelines give researchers advice on how to approach experimental design and how to report data to aid reproducibility [68–70]; many of these guidelines have been developed as part of wider activities to ensure transparency. In certain instances, these guidelines have contributed to the Minimum Information for Biological and Biomedical Investigations [71] which has, ironically, not typically included those from the NMI community. As food authenticity testing represents the most advanced example of the application of measurement science to nucleic acid analysis, there are numerous examples where associated guidance [72] and guidelines are available to support the most accurate application of molecular analysis [73–75]. As a result of the leading work that the NMIs have conducted over the last 20 years in nucleic acid analysis for foods, they are also increasingly supporting standards in other areas of molecular analysis such as biotechnology [76] and diagnostics [77]; nucleic acid analysis is also being included in standards for metrological traceability of in vitro diagnostic tests [78] due to work conducted by NAWG members. It is likely that the NAWG will continue to support an increasingly diverse range of stakeholders as the routine application of nucleic acid analysis increases.

## Metrology for food safety and authentication

Nucleic acid analysis for food authentication is most metrologically advanced (in terms of traceability and understanding sources of uncertainty), relative to other routinely applied nucleic acid measurements. Therefore, most of the NAWG-supported CMCs for NMIs exist in the food analysis sector. To date (as of September 2021), all completed NAWG Key Comparisons have been in the area of food analysis and related to the needs of NMIs and DIs to provide services for detection, identification, and quantification of genetically modified organisms (GMOs) and food adulterations (Table S1).

The use of GMO crops has increased significantly since the 1990s. Commercial cultivation of GMOs contributes to global feed, fiber, food, and fuel production; however, the safety of GMOs and their products is a major concern in some countries. National legislation regulating GMOs and their products varies significantly across jurisdictions. A global reference measurement system, including reference materials and reference methods, needs to be established to facilitate international trade and provide consumers with information on the levels of GMO constituents. Nucleic acid analysis methods are commonly used to quantify GMO content in processed food and feed [79, 80] and to monitor for food adulteration [75].

NAWG is working to support members in developing nucleic acid reference materials and calibration services for relative GMO quantity and food adulteration. This process involves DNA extraction from a complex biological matrix, followed by measurement of specific genomic DNA fragments—the GMO-specific sequence and the endogenous (species-specific) gene-specific sequence. The measurements are performed either by qPCR using an independent reference material as a calibrant or by dPCR [81–83].

A series of four key comparisons enabled NMIs and DIs to demonstrate and document their capabilities in measurements of the number of copies of specified intact sequence fragments extracted from different matrices and to determine their relative quantity. In the first two studies, matrices were rich in polymeric carbohydrate (amylose and amylopectin) and poor in fat: maize (*Zea maize* L.) seed powder in the CCQM-K86 study [84] and rice (*Oryza sativa* L.) seed powder in the CCQM-K86.b study [85]. In the third study, CCQM-K86.c, high-fat/oil matrix was selected, represented by rapeseed/canola (*Brassica napus* L.) seed powder [86]. Currently, a study on measuring DNA in high-protein matrix (meat) is in progress. All three studies on plant seed matrices had good agreement in the reported results, despite low relative quantities measured (ranging between 0.1 and 4% of GM content). While in the first study the majority of laboratories used the qPCR approach and calibrators for measurements, in the third study, all laboratories used the dPCR approach without calibrators.

These results confirmed consistency of measurements at NMIs and DIs worldwide, which resulted in the establishment of Calibration and Measurement Capabilities (CMCs) in 7 NMIs/DIs at present (Table S2).

## Metrology for health

While molecular testing is used in clinical diagnostics, reference methods are not as prevalent in this sector as they are in clinical chemistry; however, due to the desire of stakeholders to apply increasingly advanced high-throughput and quantitative measurements, this is changing and NAWG activities in the health sector are increasing. Currently, molecular diagnostic tools are used to genotype patients, to quantify pathogenic burden, to quantify transcriptional surrogate biomarkers of disease, or to measure disease predictors using subtle changes at the epigenetic level. To support NMI measurement capacity in these areas, a series of pilot studies focused on the identification and quantification of nucleic acid sequences were conducted (Table S3). In accordance with the BIPM guidelines measurement comparisons in CIPM MRA (CIPM MRA-G-11) [87], pilot studies are a third category of comparisons typically conducted to establish measurement parameters for a “new” field or instrument or as a training exercise.

To date, health-related NAWG pilot studies have mainly focused on the relative or absolute quantification of specific targets in simple matrices (aqueous buffer or cell suspension). The studies used a variety of sample types including pure synthetic and natural RNA and DNA, as well as whole cells.

The initial health studies aimed to investigate the capabilities to support measurements of mRNA biomarkers. Two studies were organized using exogenous synthetic targets from the External RNA Controls Consortium (ERCC) [33] as measurands. For the CCQM-P103 study, ERCC number 81 was selected as the measurand, while in the subsequent CCQM-P103.1 study, six ERCC standards were measured along with three endogenous gene transcripts present in the human cell line RNA background [88]. In the latter study, the majority of participants used RT-qPCR to measure the transcripts, while two participants used RT-dPCR and one used RNA-Seq. The results of these studies showed that the participating laboratories were able to measure target RNA quantities and fractional abundance (ratios) in a complex (total RNA) background with good concordance. In addition, the study highlighted the lack of a standardized approach for uncertainty calculations, especially when performing fractional abundance quantifications. Based on these results, a multiple cancer cell biomarker measurement pilot study (CCQM-P155) was organized, which used whole cell materials for analysis, to enable participant laboratories to develop and compare their nucleic acid extraction capabilities (Table S3). Another study dedicated to supporting the DNA-based diagnostics of cancer (CCQM-P184) is ongoing. The aim of this study is to measure the copy number concentrations of a single nucleotide variant (SNV) and small insertions and deletions (INDEL) present in a background of wild-type (wt) DNA (Table S3). To expand the measurement capabilities from small variants to large (structural) variants, a new study (CCQM-K176) on copy number variation of a breast cancer biomarker (HER2) is being conducted; this is the first key study in cancer diagnostics.

Following the progress in the field and to further explore the potential for using higher-accuracy SI-traceable methods for nucleic acid quantification, a study on absolute quantification of HIV-1 RNA copy number (CCQM-P199) was proposed followed by a similar study on SARS-CoV-2 copy number quantification (CCQM-P199.b) which was NAWG’s response to the COVID-19 pandemic. Both studies demonstrated that NAWG members are capable of high-accuracy measurement of RNA molecules in buffered solutions. The CCQM-P199.b study demonstrated SARS-CoV-2 quantification was possible with most laboratories submitting values with ± 40% of the mean [45]. Reports of these studies are in preparation. These five RNA pilot studies open the possibility of future key comparisons, which are selected by the corresponding CCs to test the principal techniques and methods in the field [87].

In addition to studies organized by NAWG, NMIs and DIs are establishing their measurement capacities in support of different health areas within their regional metrology organizations. Within the aforementioned projects (Infect-Met, AntiMicroResist, BioSITrace, and SEPTIMET), reference measurement procedures for nucleic acid measurements were or are being developed. One of the aims of the BioSITrace project was to establish dPCR as a primary reference measurement procedure for DNA copy number quantification through comprehensive evaluation of sources of error and comparison with mass spectrometry measurements. A frequently occurring mutation in colorectal cancer (KRAS G12D) was used as a model, and the accuracy of dPCR for copy number quantification of point mutations was assessed by evaluating potential sources of uncertainty influencing measurements [89]. This work offered further evidence on the accuracy of dPCR measurements and supported its potential as a SI-traceable method. The measurement procedure developed within BioSITrace became the first reference measurement procedure for nucleic acids listed in the database of JCTLM [13]. Results of this project have contributed also to the revision of ISO 17511:2020 in vitro diagnostic medical devices — requirements for establishing metrological traceability of values assigned to calibrators, trueness control materials, and human samples [78].

Similarly in the frame of Infect-Met and AntiMicroResist projects, methods for reliable quantification of several infectious agents, human influenza A virus, human cytomegalovirus, *Mycobacterium tuberculosis*, and HIV, and their resistance were investigated [90–97]. One of the measurement procedures developed and evaluated in these projects, namely quantification of DNA extracted from human cytomegalovirus, has been also listed in the JCTLM database. This measurement procedure has been evaluated in External Quality Assessment schemes and showed the potential to harmonize for routine hCMV load testing [98]. The SEPTIMET project is currently developing quantitative methods and potential reference materials, as metrological support is needed for rapid near patient testing and innovative methods. In the response to the pandemic, part of the research within the SEPTIMET has been dedicated to SARS-CoV-2–related issues [99].

Besides the development of accurate measurement procedures, NMIs and DIs are developing controls and reference materials to support both local and international health sectors. For example, the National Institute of Standards and Technology (NIST) offers a range of reference materials for biometrology, including materials for cancer measurements [100–102], viral pathogens [103], microbial DNA (https://www-s.nist.gov/srmors/view_detail.cfm?srm=8375), and human DNA [104, 105]. These materials cover a range of applications from relative abundance (SRM 2373) to absolute copy number (SRM 2365) to NGS (RM 8392). Similarly, the National Institute of Metrology, China (NIM China) has developed different DNA reference materials such as KRAS mutations in genomic DNA [106], BRAF V600E mutation, and different EGFR variants mimicking circulating tumor DNA [107, 108]. The Korea Research Institute of Standards and Science, South Korea (KRISS) has developed a representative DNA mixture in serum CRM for non-invasive prenatal testing (NIPT). This CRM is formulated to closely mimic the serum sample from a pregnant woman bearing a fetus with trisomy 21, the most common chromosomal abnormality. In a pregnant woman’s serum, a fraction of fetal DNA is present in addition to her own DNA, which are the target analytes in NIPT. The KRISS CRM for NIPT achieves its measurement traceability to the exact DNA amount mixed into the DNA-free serum matrix [109].

In response to the COVID-19 pandemic in 2020, a number of NMIs and DIs have developed SARS-CoV-2-related reference materials (RM), aiming to support nucleic acid testing [110]. Most of these RMs are in vitro transcribed RNA of SARS-CoV-2 genes [111]. The list of institutes offering materials made from in vitro transcripts of SARS-CoV-2 includes NIM China [110]; NIST (USA) [110]; the National Measurement Institute (NMI Australia), Department of Industry, Science, Energy and Resources, Australian Government [112]; KRISS (South Korea) [110]; and TÜBİTAK UME (Turkey) [113]. Additionally, KRISS (South Korea), NIBSC, and NIM China have developed another form of RM, which is packaged SARS-CoV-2 RNA [110, 111, 114]. A few NMIs and DIs participated in the Collaborative Study for the Establishment of a WHO International Standard for SARS-CoV-2 RNA containing whole virus [115, 116]. These materials have provided reliability in SARS-CoV-2 nucleic acid testing in local and international communities.

## Future perspectives

As we progress into the next decade, NAWG activities will likely continue to support food-associated testing with key comparisons reflecting additional unmet needs, such as different matrices (e.g., protein rich), species (e.g., food adulterations), and molecular challenges (e.g., fragment size, strandedness, modifications like methylation). It is anticipated that food-associated measurements will expand to the agriculture/biotechnology sector; studies to support the monitoring of crop disease are planned which will expand the capacities of NAWG in view of the One Health approach. In line with its strategy heading for 2030 as well as the CCQM strategy document 2021–2030 [43], NAWG will considerably strengthen its activities associated with molecular reference measurement procedures (including structural and sequence purity) and reference materials to support clinical nucleic acid analysis. These activities are likely to build on established capabilities for underpinning SI-traceable quantitative measurements of nucleic acid copy number (per unit volume) in aqueous solutions [89, 91] and explore routes to apply such capacity to assist in matrix reference materials and/or reference measurements on real samples [90, 96, 117].

Developed capacities will also be of value to the other sectors such as industrial biotechnology where novel methods, such as CRISPR CAS-9–based genetic modification, are being applied [118, 119]. Biotechnology, especially the biopharmaceutical industry, performs numerous measurements to ensure the safety, quality, and efficiency of manufactured products [120]. Well-defined procedures for nucleic acid analysis are listed in almost all national and international pharmacopeias (e.g., IP, EP, USP, BP, JP, RSP, and others). NAWG member NMIs and DIs are establishing a solid base for measurements performed in industrial bioanalytical laboratories, by providing CRMs or reference measurement services to harmonize measurement results. As an example, measurement capabilities on the quantification of mycoplasmas, a common biotechnology manufacturing contaminant, will be investigated in an ongoing NAWG pilot study.

The increasing need for genomic, transcriptomic, and epigenomic analyses means that the NAWG will need to explore the development of strategies to support advanced sequencing capabilities, including for purity analysis. The metrological support in this area is lacking [120], and challenges associated with these types of “non-targeted” methods are likely to increase with the development of newer, simplified sequencing technologies more suitable to less specialized settings.

Last but not least, DNA measurements have become a valuable tool for environmental biodiversity monitoring. Sequencing of environmental DNA (DNA isolated from environmental samples) allows the identification of bacterial and eukaryotic species. Environmental DNA measurements, including quantitative DNA analysis, are used to study biodiversity and to monitor ecosystem changes. The NAWG strategy includes DNA (genomic and mitochondrial) sequencing–based species identification and bacterial quantification; participating NMIs/DIs will establish calibration and measurement capabilities in these areas in the near future.

## Conclusions

As the use of nucleic acid analysis is increasingly applied across a wide range of sectors, measurement science will facilitate more a rapid and efficiency application. This will be needed to:

1. Increase research accuracy and therefore our understanding of the biological systems we study
2. Enable the translation of findings to routine application through preempting considerations that will ensure test applications are both robust and reproducible
3. Facilitate routine application of nucleic acid analysis methods by enabling developers and users to demonstrate confidence in delivering the most accurate measurements

Along with associated relevant organizations, the CCQM NAWG and associated NMIs/DIs will continue to develop routes to ensure measurement accuracy and traceability for nucleic acid analysis. This will support measurement accuracy as molecular methods become increasingly applied for routine measurements to a wide variety of sectors.

## Supplementary Information

Below is the link to the electronic supplementary material.

Supplementary file1 (DOCX 37 KB)

Supplementary file2 (PDF 441 KB)

## Data Availability

Not applicable.
