# Supplementary material for: Metrological framework to support accurate, reliable, and reproducible nucleic acid measurements
Source: Anal Bioanal Chem. 2021 Nov 4;414(2):791–806. doi: 10.1007/s00216-021-03712-x (PMC8568362; doi:10.1007/s00216-021-03712-x)
Supplement: Supplementary file 1 — Supplementary file1 (DOCX 37 KB) [file 216_2021_3712_MOESM1_ESM.docx]

**Supplementary Information for**

**Metrological Framework to Support Accurate, Reliable, and Reproducible Nucleic Acids Measurements**

Mojca Milavec^1^, Megan H. Cleveland^2^, Young-Kyung Bae^3^, Robert I. Wielgosz^4^, Maxim Vonsky^5^, Jim F. Huggett^6,7^

^1^ Department of Biotechnology and Systems Biology, National Institute of biology, Večna pot 111, 1000 Ljubljana, Slovenia

^2^ National Institute of Standards and Technology, 100 Bureau Drive, Gaithersburg, Maryland 20899, United States

^3^ Korea Research Institute of Standards and Science (KRISS), Daejeon, Republic of Korea

^4^ Bureau International des Poids et Mesures (BIPM), Pavillon de Breteuil , 92312 Sèvres Cedex, France

^5^ D.I. Mendeleev Institute for Metrology, Moskovsky pr., 19, Saint-Petersburg, 190005, Russian Federation

^6^ National Measurement Laboratory (NML), LGC, Queens Road, Teddington, Middlesex, TW11 0LY, United Kingdom

^7^ School of Biosciences & Medicine, Faculty of Health & Medical Science, University of Surrey, Guildford, United Kingdom

**Corresponding author:**

Mojca Milavec, Department of Biotechnology and Systems Biology, National Institute of biology, Večna pot 111, 1000 Ljubljana, [mojca.milavec@nib.si](mailto:mojca.milavec@nib.si)

**ORCHID ID**

Mojca Milavec 0000-0002-5794-2109

Megan H. Cleveland [0000-0002-5584-9314](https://orcid.org/0000-0002-5584-9314)

Young-Kyung Bae 0000-0001-5293-8752

Robert I. Wielgosz

Maxim Vonsky [0000-0003-4061-7411](https://orcid.org/0000-0003-4061-7411)

Jim F. Huggett [0000-0002-0945-1911](https://orcid.org/0000-0002-0945-1911)

**Table of Contents**

[Table S1 Nucleic acid analysis interlaboratory comparisons in the field of health organized under CCQM NAWG and its predecessor CCQM BAWG. All conducted studies were combinations of Key Comparisons and Pilot studies, as defined in the Measurement comparisons in the CIPM MRA, Guidelines for organizing, participating and reporting CIPM MRA-G-11 [1] 3](#_Toc76288631)

[Table S3 Nucleic acid analysis interlaboratory comparisons in the field of health organized under CCQM NAWG and its predecessor CCQM BAWG. All conducted studies were Pilot studies, as defined in the Measurement comparisons in the CIPM MRA, Guidelines for organizing, participating and reporting CIPM MRA-G-11 [1] 4](#_Toc76288632)

[References 6](#_Toc76288633)

# Table S1 Nucleic acid analysis interlaboratory comparisons in the field of health organized under CCQM NAWG and its predecessor CCQM BAWG. All conducted studies were combinations of Key Comparisons and Pilot studies, as defined in the Measurement comparisons in the CIPM MRA, Guidelines for organizing, participating and reporting CIPM MRA-G-11 [1]

| Study ID | Title of the study | Coordinating Laboratory | Number of participants | Start year | Short description |
| --- | --- | --- | --- | --- | --- |
| CCQM-K86 and CCQM-P113.1 | Relative quantification of genomic DNA fragments extracted from a biological tissue | JRC | 13 | 2010 | Analytes: MON810 specific sequence and maize specific sequence  Measurands: copy number concentration ratio of MON810 specific sequence and maize specific sequence  Matrix: powdered seed  Methods: DNA extraction, qPCR  Status: Published [2] |
| CCQM-K86.b and CCQM-P113.3 | Relative quantification of Bt63 in GM rice matrix sample | NIMC/GLHK | 9 | 2015 | Analytes: Bt63-specific sequence and rice specific sequence *REB4*  Measurands: copy number concentration ratio of Bt63 specific sequence and rice specific sequence *REB4*  Matrix: rice powder  Methods: DNA extraction, qPCR, dPCR  Status: Published [3] |
| CCQM-K86.c and CCQM-P113.4 | Relative quantification of genomic DNA fragments extracted from high oil matrix (OSR/canola) | NRC/JRC | 12 | 2016 | Analytes: of DP-Ø73496-4 specific sequence, rapeseed *FatA(A)* specific sequence, GT73/RT73 specific sequence, rapeseed *FatA* specific sequence.  Measurands: copy number concentration of DP-Ø73496-4 specific sequence and rapeseed specific sequence *FatA(A)* and their ratio and copy number concentration of GT73/RT73 specific sequence and rapeseed specific sequence *FatA* and their ratio  Matrix: rapeseed powder  Methods: DNA extraction, dPCR  Status: Published [4] |
| CCQM-K86.d and CCQM-P113.5 | DNA Ratio in a High Protein Matrix:  Relative quantification and fractional abundance of genomic DNA  extracted from a biological tissue | NIMT/TUBITAK UME |  | 2021 | In progress |

# Table S3 Nucleic acid analysis interlaboratory comparisons in the field of health organized under CCQM NAWG and its predecessor CCQM BAWG. All conducted studies were Pilot studies, as defined in the Measurement comparisons in the CIPM MRA, Guidelines for organizing, participating and reporting CIPM MRA-G-11 [1]

| Study ID | Title of the study | Coordinating Laboratory | Number of participants | Start year | Short description |
| --- | --- | --- | --- | --- | --- |
| CCQM-P94 | Quantification of DNA methylation | KRISS | 5 | 2006 | Analyte: 100-bp PCR products with defined numbers of methyl cytosines  Measurands: relative quantity of methylated cytosine bases in PCR products  Matrix: buffered solution  Methods: dNMPs following enzymatic hydrolysis of DNA (using HPLC/CE/Other)  Status: Published [5] |
| CCQM-P103 | Quantification of RNA transcript | NML at LGC | 8 | 2007 | Analyte: ERCC 81 specific sequence  Measurand: copy number concentration of ERCC 81  Matrix: buffered solution  Method: RT-qPCR  Status: Internal report |
| CCQM-P94.1 | Quantitative analysis of methylation status of CDKN2 A gene in genomic DNA matrix | KRISS | 6 | 2008 | Analyte: unmethylated and methylated CDKN2A (P16)  Measurands: relative quantity of methylated cytosine bases in CDKN2 A gene  Matrix: buffered solution  Methods: CE, sequencing, HPLC, UPLC  Status: Internal report |
| CCQM-103.1 | Measurement of multiple RNA transcripts | NML at LGC | 13 | 2011 | Analytes: ERCC -00013, -00025, -00042, -00099, -00113 and -00171; mRNA of [matrix metallopeptidase](https://www.sciencedirect.com/topics/medicine-and-dentistry/matrix-metalloproteinase) 1, [nestin](https://www.sciencedirect.com/topics/medicine-and-dentistry/nestin) and [solute carrier family](https://www.sciencedirect.com/topics/agricultural-and-biological-sciences/solute-carrier-family) 1 (glial high affinity glutamate transporter) member 3 specific sequences.  Measurands: copy number concentrations of ERCC -00013, -00025, -00042, -00099, -00113 and -00171; [matrix metallopeptidase](https://www.sciencedirect.com/topics/medicine-and-dentistry/matrix-metalloproteinase) 1, [nestin](https://www.sciencedirect.com/topics/medicine-and-dentistry/nestin), and [solute carrier family](https://www.sciencedirect.com/topics/agricultural-and-biological-sciences/solute-carrier-family) 1 (glial high affinity glutamate transporter) member 3 and their ratios  Matrix: buffered solution  Methods: RT-qPCR, RT-dPCR, RNA Seq  Status: Published [6] |
| CCQM-P94.2 | Quantification of DNA methylation | KRISS |  | 2012 | Analyte: CDKN2 A gene  Measurands: relative quantity of methylated cytosine bases in CDKN2 A gene  Matrix: buffered solution  Methods: liquid chromatography-isotope dilution mass spectrometry( HPLC-IDMS) and Sanger sequencing method( SS)  Status: Internal report, publication in preparation |
| CCQM-P155 | Multiple cancer cell biomarker measurement | NML at LGC | 10 | 2015 | Analyte: KRT19 (Type I), Trefoil Factor 1, TATA Binding Protein mRNA specific sequences  Measurands: copy number concentrations of breast cancer mRNA biomarkers (KRT19 (Type I) and Trefoil Factor 1) and mRNA of a reference gene TATA Binding Protein and their ratios  Matrix: whole cell materials  Methods: NA extraction, RT-qPCR, RT-dPCR  Status: Internal report, publication in preparation |
| CCQM-P184 | Copy number concentration and fractional abundance of a mutation (SNV or INDEL) mixed with WT DNA | NMIA/NML at LGC | 12 | 2017 | Analytes: BRAF 1799A ,BRAF 1799T, EGFR exon 19 delta 746-750, EGFR specific sequences  Measurands: copy number concentration of the BRAF single nucleotide variant 1799A and the BRAF reference sequence 1799T and their ratio; copy number concentration of human EGFR exon 19 deletion variant (delta746-750) and copy number concentration of human wild-type EGFR and their ratio  Matrix: buffered solution  Method: dPCR  Status: Internal report in preparation |
| CCQM-P199 | Copy number concentration of HIV-1 RNA genomic sequences | NML at LGC/(NIBSC) | 13 | 2018 | Analytes: HIV-1 *gag* gene RNA specific sequence  Measurand: copy number concentration of *gag* gene  Matrix: buffered solution  Methods: RT-dPCR, flow cytometry, isotope dilution mass spectrometry  Status: Internal report in preparation |
| CCQM-P199b | Copy number concentration of SARS-CoV-2 RNA genomic sequences | NML at LGC/NIMC/NIBSC/NIST | 21 | 2020 | Analytes:SARS-CoV-2 *N* gene and *E* gene RNA specific sequences  Measurands: copy number concentration of *N* and/or *E* gene  Matrix: buffered solution  Methods: RT-dPCR, flow cytometry, isotope dilution mass spectrometry  Status: Internal report in preparation, preliminary data available [7] |

# References

1. CIPM MRA documents. https://www.bipm.org/en/cipm-mra/cipm-mra-documents. Accessed 20 Jun 2021

2. Corbisier P, Vincent S, Schimmel H, Kortekaas A-M, Trapmann S, Burns M, Bushell C, Akgoz M, Akyürek S, Dong L, Fu B, Zhang L, Wang J, Pérez Urquiza M, Bautista JL, Garibay A, Fuller B, Baoutina A, Partis L, Emslie K, Holden M, Chum WY, Kim H-H, Phunbua N, Milavec M, Zel J, Vonsky M, Konopelko LA, Lau TLT, Yang B, Hui MHK, Yu ACH, Viroonudomphol D, Prawettongsopon C, Wiangnon K, Takabatake R, Kitta K, Kawaharasaki M, Parkes H (2012) CCQM-K86/P113.1: Relative quantification of genomic DNA fragments extracted from a biological tissue. Metrologia 49:08002–08002 . https://doi.org/10.1088/0026-1394/49/1A/08002

3. Dong L, Sui Z, Wang J, Tang VHM, Chum WWY, Lee F, Sin DWM, Pérez–Urquiza M, Burns M, Ellison SLR, Parkes H, Milavec M, Prawettongsopon C, Griffiths KR, McLaughlin JLH, Shibayama S, Akyurek S, Akgoz M (2018) Final report for CCQM-K86.b relative quantification of Bt63 in GM rice matrix sample. Metrologia 55:8017 . https://doi.org/10.1088/0026-1394/55/1a/08017

4. Mester Z, Corbisier P, Ellison SLR, Gao Y, Niu C, Tang V, Lee F, Pérez-Urquiza M, Suárez AR, Burns M, Milavec M, Wiangnon K, Griffiths KR, McLaughlin JLH, Shibayama S, Takatsu A, Akgoz M, Vonsky M, Runov A, Guerrero JEL (2020) Final report of CCQM-K86.c. Relative quantification of genomic DNA fragments extracted from a biological tissue. Metrologia 57:08004–08004 . https://doi.org/10.1088/0026-1394/57/1A/08004

5. Yang I, Kim SK, Burke DG, Griffiths K, Kassir Z, Emslie KR, Gao Y, Wang J, Foy CA, Pardos-Pardos AC, Ellison S, Domann PJ, Fujii SI, Park SR (2009) An international comparability study on quantification of total methyl cytosine content. Anal Biochem 384:288–295 . https://doi.org/10.1016/j.ab.2008.09.036

6. Devonshire AS, Sanders R, Whale AS, Nixon GJ, Cowen S, Ellison SLR, Parkes H, Pine S, Salit M, McDaniel J, Munro S, Lund S, Matsukura S, Sekiguchi Y, Kawaharasaki M, Granjeiro JM, Falagan-Lotsch P, Saraiva AM, Couto P, Yang I, Kwon H, Park SR, Demšar T, Žel J, Blejec A, Milavec M, Dong L, Zhang L, Sui Z, Wang J, Viroonudomphol D, Prawettongsopon C, Partis L, Baoutina A, Emslie K, Takatsu A, Akyurek S, Akgoz M, Vonsky M, Konopelko L, Cundapi EM, Urquiza MP, Huggett JF, Foy CA (2016) An international comparability study on quantification of mRNA gene expression ratios: CCQM-P103.1. Biomol Detect Quantif 8:15–28 . https://doi.org/10.1016/j.bdq.2016.05.003

7. National Measurement Institutes demonstrate high accuracy reference measurement system for SARS-CoV-2 testing. https://www.bipm.org/en//-/2020-nmi-covid. Accessed 20 Jun 2021
