# Supplementary material for: Metrological framework to support accurate, reliable, and reproducible nucleic acid measurements
Source: Anal Bioanal Chem. 2021 Nov 4;414(2):791–806. doi: 10.1007/s00216-021-03712-x (PMC8568362; doi:10.1007/s00216-021-03712-x)
Supplement: Supplementary file 2 — Supplementary file2 (PDF 441 KB) [file 216_2021_3712_MOESM2_ESM.pdf]

Mojca Milavec[1], Megan H. Cleveland[2], Young-Kyung Bae[3], Robert I. Wielgosz[4], Maxim Vonsky[5], Jim F. Huggett[6,7]

[1] Department of Biotechnology and Systems Biology, National Institute of biology, Večna pot 111, 1000 Ljubljana, Slovenia
[2] National Institute of Standards and Technology, 100 Bureau Drive, Gaithersburg, Maryland 20899, United States
[3] Korea Research Institute of Standards and Science (KRISS), Daejeon, Republic of Korea
[4] Bureau International des Poids et Mesures (BIPM), Pavillon de Breteuil , 92312 Sèvres Cedex, France
[5] D.I. Mendeleev Institute for Metrology, Moskovsky pr., 19, Saint-Petersburg, 190005, Russian Federation
[6] National Measurement Laboratory (NML), LGC, Queens Road, Teddington, Middlesex, TW11 0LY, United Kingdom
[7] School of Biosciences & Medicine, Faculty of Health & Medical Science, University of Surrey, Guildford, United Kingdom

Corresponding author:
Mojca Milavec, Department of Biotechnology and Systems Biology, National Institute of biology, Večna pot 111, 1000 Ljubljana,
mojca.milavec@nib.si
ORCHID ID
Mojca Milavec 0000-0002-5794-2109
Megan H. Cleveland 0000-0002-5584-9314
Young-Kyung Bae 0000-0001-5293-8752
Robert I. Wielgosz
Maxim Vonsky 0000-0003-4061-7411
Jim F. Huggett 0000-0002-0945-1911

Table S2 Calibration and Measurement Capabilities (CMCs) on Measurement Service Category Food, Subcategory GMOs published in the BIPM Key Comparisons Database

Chemistry and Biology

Data copy established on 14 April 2021
Food
China, NIM (National Institute of Metrology)
Hong Kong, China, GL (Government Laboratory)
Mexico, CENAM (Centro Nacional de Metrologia)
Russian Federation, VNIIM (D.I. Mendeleyev Institute for Metrology, Rosstandart)
Slovenia, MIRS/NIB/FITO (MIRS/National Institute of Biology/Department of Biotechnology and Systems Biology)
Turkey, UME (TÜBITAK Ulusal Metroloji Enstitüsü)
United Kingdom, LGC (Laboratory of the Government Chemist)

Country code	Institute	Meas. Serv. Category	Meas. Serv. Sub-Category	Matrix	Analyte or component	Quantity	CMC Minimum value	CMC Maximum value	Unit	CMC Expanded Uncertainty Minimum value	CMC Expanded Uncertainty Maximum value	Unit	Coverage factor	Level of Confidence in %	Type of uncertainty	Uncertainty convention	Mecanism for measurement service delivery	Comments	Approval date	NMI service identifier
SI	MIRS/NIB/FITO	Food	GMOs	raw or not highly processed ground maize material	Genomic DNA. Copy number ratio of specified intact sequence fragments of 50 to 150 nucleotides length in a single genomic DNA extract.	DNA copy number ratio	0.5	2.1	%	0.2	0.5	%	2.0	95.0	Absolute	One	Value assignment to customer supplied samples	The exact term for the "Matrix or material" is "Raw or not highly processed ground maize material, maize seed and grains and other plant derived seed and grain materials of similar matrix complexity in terms of protein, carbohydrate and lipid composition"	2017-06-30	FITO002
SI	MIRS/NIB/FITO	Food	GMOs	raw or not highly processed ground soya material	Genomic DNA. Copy number ratio of specified intact sequence fragments of 50 to 150 nucleotides length in a single genomic DNA extract.	DNA copy number ratio	0.5	2.1	%	0.2	0.5	%	2.0	95.0	Absolute	One	Value assignment to customer supplied samples	The exact term for the "Matrix or material" is "Raw or not highly processed ground soya material, soya seed and grains and other plant derived seed and grain materials of similar matrix complexity in terms of protein, carbohydrate and lipid composition"	2017-06-30	FITO003
GB	LGC	Food	GMOs	ground soya material, soya seed material and other plant derived seed material	Genomic DNA. Copy number ratio of specified intact sequence fragments of 70 to 150 nucleotides length in a single genomic DNA extract	DNA copy number ratio	0.12	3.5	%	0.05	1.11	%	2.0	95.0	Absolute	One	Value assignment of client samples	The exact term for the "Matrix or material" is ground soya material, soya seed material and other plant derived seed materials of similar matrix complexity in terms of protein, carbohydrate and lipid composition	2015-07-08	Bio-003
GB	LGC	Food	GMOs	ground maize material, maize seed material and other plant derived seed material	Genomic DNA. Copy number ratio of specified intact sequence fragments of 70 to 150 nucleotides length in a single genomic DNA extract	DNA copy number ratio	0.12	3.5	%	0.05	1.11	%	2.0	95.0	Absolute	One	Value assignment of client samples	The exact term for the "Matrix or material" is ground maize material, maize seed material and other plant derived seed materials of similar matrix complexity in terms of protein, carbohydrate and lipid composition	2015-07-08	Bio-004
GB	LGC	Food	GMOs	raw, high starch ground seed materials and grains	specified intact sequence fragments of a length up to 150 nucleotides in a single genomic DNA extract	Copy number ratio	0.88	3.05	%	0.35	1.3	%	2.0	95.0	Absolute	Two	Value assignment of client samples		2018-06-28	Bio-007
TR	UME	Food	GMOs	raw or not highly processed maize seed material	Genomic DNA. Copy number ratio of specified intact sequence fragments of 60 to 150 nucleotides length in a single genomic DNA extract	DNA copy number ratio	0.7	5.5	%	0.2	1.7	%	2.0	95.0	Absolute	One	Value assignment of client samples	The exact term for the "Matrix or material" is "raw or not highly processed maize seed material and other seed materials with similar level of matrix complexity"	2017-06-30	G3BA-1100
TR	UME	Food	GMOs	unprocessed, high starch ground seed matrix material […]	[…]specified intact sequence fragments of a length up to 150 nucleotides […]	DNA copy number ratio	0.5	3.0	%	0.2	0.5	%	2.0	95.0	Absolute	One	Value assignment of client samples	The exact term for the "Analyte or component" is "quantification of the ratio of the number of copies of specified intact sequence fragments of a length up to 150 nucleotides following extraction from an unprocessed, high starch ground seed matrix. with a copy number ratio from 0.005 to 1". The exact term for the "Matrix or material" is "unprocessed, high starch ground seed matrix material and other seed materials with similar level of matrix complexity"	2018-06-28	G3BA-1100
CN	NIM	Food	GMOs	raw maize	copy number ratio of specified intact sequence fragments of 70 to 120 nucleotides length in a single genomic DNA extract	DNA copy number ratio of two DNA fragments with specified sequence	0.3	2.5	%	25.0	25.0	%	2.0	95.0	Absolute	Two	Value assignment of sample in PT scheme	The exact term for the "Analyte or component" is "Quantification of the ratio of the number of copies of specified intact sequence fragments of a length in the range of 70 to 120 nucleotides in a single genomic DNA extract from biological materials"	2014-11-03	NIM Food(B)-3
CN	NIM	Food	GMOs	raw, high starch ground seed materials and grains	specified intact sequence fragments of a length up to 150 nucleotides length in a single genomic DNA extract	Copy number ratio [Exogenous sequence/ endogenous sequence]	0.88	3.05	%	0.08	0.34	%	2.0	95.0	Absolute	Two	CRM value assignment and verification,value assignment of client samples	Copy number ratio [Exogenous sequence/ endogenous sequence]	2018-09-26	Food-232
HK	GL	Food	GMOs	raw (or not highly processed) maize seed material and grains	specified intact sequence segments of a length in the range of 60 to 150 nucleotides in a single genomic DNA extract	Copy number ratio	0.22	2.38	%	0.08	0.85	%	2.0	95.0	Absolute	Two	Value assignment of client samples		2016-10-03	GLHK054
HK	GL	Food	GMOs	raw, high starch ground seed materials and grains	specified intact sequence fragments of a length up to 150 nucleotides length in a single genomic DNA extract	Copy number ratio	0.88	3.05	%	0.18	0.61	%	2.0	95.0	Absolute	Two	Value assignment of client samples		2018-09-26	GLHK083
RU	VNIIM	Food	GMOs	high oil/fat matrix material and other material with the same level of complexity	Ratio of the number of copies of specified intact sequence fragments of a length up to 150 nucleotides following extraction from a high fat/oil matrix, with a copy number ratio from 0.001 to 1	DNA copy number ratio	0.1	50.0	%	0.026	12.0	%	2.0	95.0	Absolute	Two	Value assignment of client samples		2019-10-23	11.3-01
GB	LGC	Food	GMOs	unprocessed, high oil/fat ground seed matrix and any other material with the same level of complexity	[…]specified intact sequence fragments of a length up to 150 nucleotides […]	DNA copy number ratio	0.001	1.0	%	0.04	0.3	%	2.0	95.0	Absolute	Two	Value assignment of client samples	The exact term for the "Analyte or component" is "Quantification of the ratio of the number of copies of specified intact sequence fragments of a length up to 150 nucleotides following extraction from an unprocessed, high oil/fat ground seed matrix, with a copy number ratio from 0.001 to 1"	2019-10-15	Bio-008
MX	CENAM	Food	GMOs	ground seed materials and grains of similar matrix complexit , in terms of protein, high starch and high oil content	specified intact sequence fragments of a length up to 150 nucleotides in a single genomic DNA extract	Copy number ratio	0.14	90.0	%	59.0	4.0	%	2.0	95.0	Absolute	Two	Value assignment of client samples		2019-10-23	Bio-001
TR	UME	Food	GMOs	unprocessed, high oil/fat ground seed matrix material and any other material with the same level of complexity	Genomic DNA. Copies of specified intact sequence fragments of a length up to 150 nucleotides from an unprocessed, high fat/oil ground seed matrix	DNA copy number ratio	0.1	100.0	%	15.0	15.0	%	2.26	95.0	Absolute	Two	Value assignment of client samples		2020-10-22	G3BA-1200
